# Overdiagnosing giant bullous emphysema as metastatic adenocarcinoma: a case report

**DOI:** 10.1186/s13019-024-03112-z

**Published:** 2024-10-01

**Authors:** Jiyun Lee, Eunsu Park

**Affiliations:** 1 Department of Thoracic and Cardiovascular Surgery, Incheon St. Mary’s Hospital, College of Medicine, The Catholic University of Korea, Seoul, Republic of Korea; 2 Department of Pathology, Incheon St. Mary’s Hospital, College of Medicine, The Catholic University of Korea, Seoul, Republic of Korea

**Keywords:** Overdiagnosis, Emphysema, Lung cancer, Adenocarcinoma, Case report

## Abstract

**Background:**

Giant bullous emphysema is characterized by large bullae occupying at least one-third of the hemithorax and leading to compression of the surrounding lung parenchyma. Overdiagnosis can occur because of the atypical appearance of hyperplastic type II pneumocytes, which may be mistaken for malignant cells.

**Case presentation:**

A 48-year-old male with a history of smoking and occupational exposure presented with dyspnea and drowsiness. Initial chest X-ray revealed a tension pneumothorax, and subsequent chest CT revealed extensive bullous emphysema and lung cancer in the right middle lobe (RML). Pathologic examination initially indicated resected bullae to metastatic adenocarcinoma, but upon review, it was determined that the reactive alveolar cells were misdiagnosed as malignant.

**Conclusions:**

This case emphasizes the need for thorough histopathological assessment and prudent interpretation of atypical cellular morphology.

## Background

Giant bullous emphysema was first described in 1937. This condition is rare and characterized by the presence of large bullae occupying at least one-third of a hemithorax that leads to compression of the surrounding lung parenchyma [1]. Patients afflicted with giant bullous emphysema typically have a history of cigarette smoking and COPD [2, 3]. Presenting features include progressive dyspnea, declining exercise tolerance, hypoxia, and decreased breath sounds. Disease progression can lead to pneumothorax, subcutaneous emphysema, and respiratory failure with hypoxia and hypercapnia [3, 4]. Giant bullous emphysema can closely resemble pneumothorax, and chest CT is the optimal method for distinguishing between the two conditions, making it the gold standard for differential diagnosis [5].

The alveoli are usually lined by type I pneumocytes but can be populated by hyperplastic type II pneumocytes under certain conditions such as diffuse alveolar damage, radiation or drug-related toxicity, infection, and inflammatory or fibrosing conditions. This atypical appearance can be quite striking and is described as “too atypical to be malignant” [6].

Herein is the report of a case of overdiagnosing metastatic adenocarcinoma as giant bullous emphysema in a 48-year-old male, written according to CARE case report guidelines.

## Case presentation

**Fig. 1:**
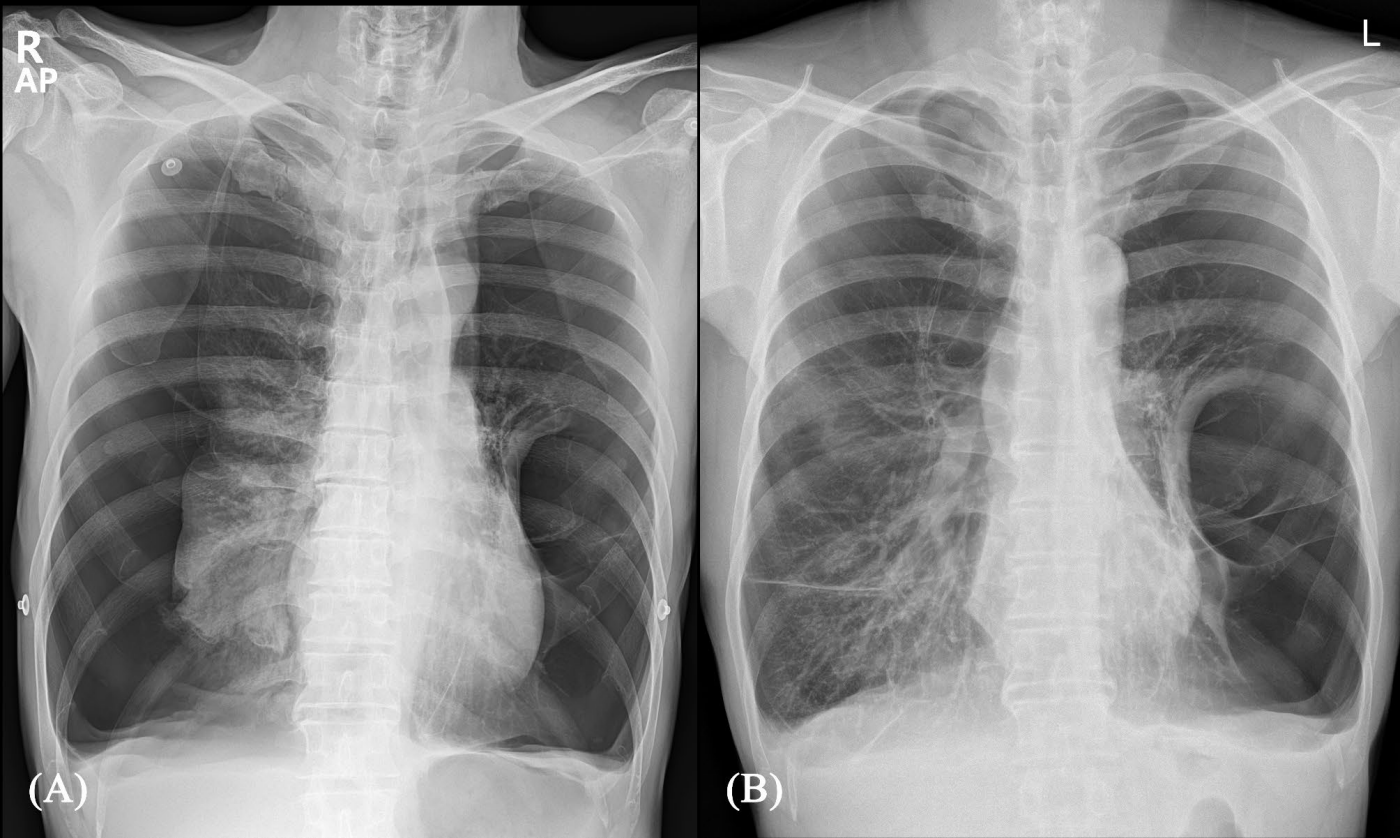
**(A)** Chest X-ray taken upon the patient’s arrival at the emergency room showing tension pneumothorax on the right side. **(B)** The most recent outpatient clinic chest X-ray before the development of pneumothorax revealed bullous emphysema

The patient, a 48-year-old male with a height of 169 cm and weight of 54 kg, presented to the emergency room with dyspnea and drowsiness, with an oxygen saturation of 50%. A chest X-ray revealed a tension pneumothorax in the right hemithorax (Fig. 1), prompting an emergency closed thoracostomy with a 24Fr chest tube.

The patient had a history of coughing, sputum production, and shortness of breath with an ECOG score of 2 for the past 2 years. He had sought medical attention from a pulmonologist and was awaiting further outpatient evaluation following chest CT and PFT. The patient had a 30-year history of smoking and occupational exposure due to working in a tunnel.

**Fig. 2:**
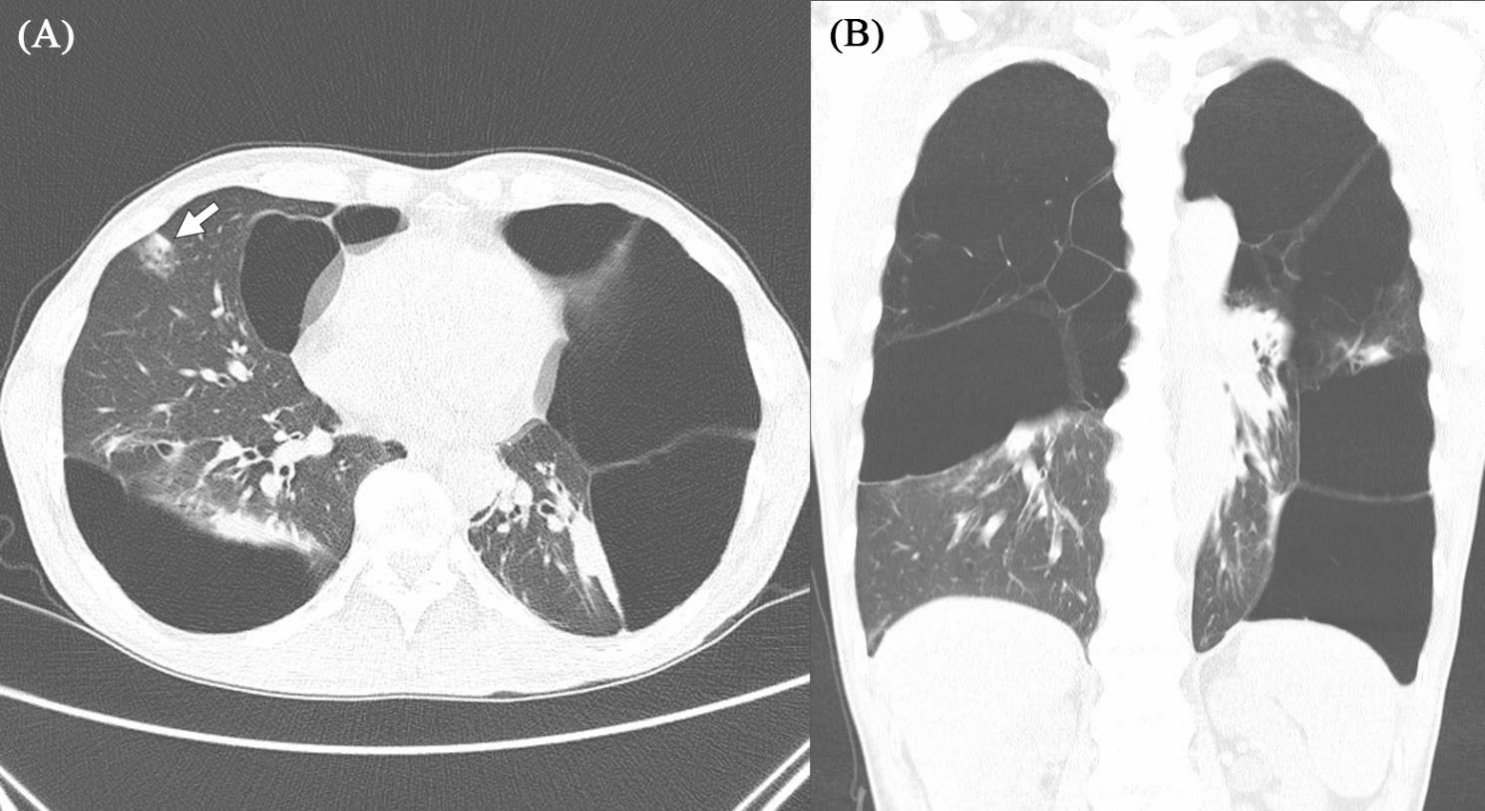
Preoperative contrast-enhanced chest CT revealed the following: **(A)** Giant bullae in both the hemithoraces and lung cancer in the right middle lobe (white arrow) in the axial view. **(B)** Giant bullous emphysema in both the hemithoraces in the coronal view. CT, computed tomography

Chest CT showed signs of extensive bullous emphysema in both lungs and a pulmonary nodule, which was suspected of being lung cancer or organizing pneumonia in the RML (Fig. 2). The PFT results revealed a significantly reduced lung function indicative of a severe obstructive pattern as follows: FVC 1.92 L 44% (postbronchodilator: 2.08 L 47%), FEV1 0.72 L 22% (postbronchodilator: 0.81 L 25%), FEV1/FVC 37% (postbronchodilator: 39%), and DLCO 7.1 mL/mmHg/min 35%. PET-CT revealed a possibly malignant tumor in the RML with a SUVmax of 3.5. Blood tests revealed an alpha-1 antitrypsin level of 129 mg/dL (reference range: 90–200) and an elevated CEA level of 7.96 ng/mL (reference range: 0–5).

Based on the findings and the suspicion of lung cancer, a multidisciplinary team recommended limited surgery for lung cancer and bullectomy. During the surgical procedure, bullae in the RUL, RML, and RLL of the lung were discovered, with most of them concentrated in the RUL. The pulmonary nodule in the RML was subjected to wedge resection and frozen section analysis, which revealed adenocarcinoma. Mediastinal lymph node dissection was also performed. Giant bullae were removed through multiple wedge resections using PTFE-reinforced endostaplers. The patient underwent chemical pleurodesis using Abnova Viscum-F four times to address the persistent air leakage. The chest tubes were removed on POD 20, and the patient was discharged on POD 21.

Pathologic examination revealed the presence of invasive adenocarcinoma with papillary (90%) and micropapillary (10%) features, which were moderately differentiated and measured 1.3 × 1.0 × 0.8 cm. The cancer had invaded the visceral pleural surface and had metastasized to the lower paratracheal lymph node. Additionally, metastatic adenocarcinoma was found in the bullae removed by bullectomy, resulting in a diagnosis of pT2aN2aM1a (AJCC 9th edition) lung cancer. The patient is currently receiving systemic chemotherapy, the dyspnea has resolved, and the PFT results have improved compared with the preoperative findings.

**Fig. 3:**
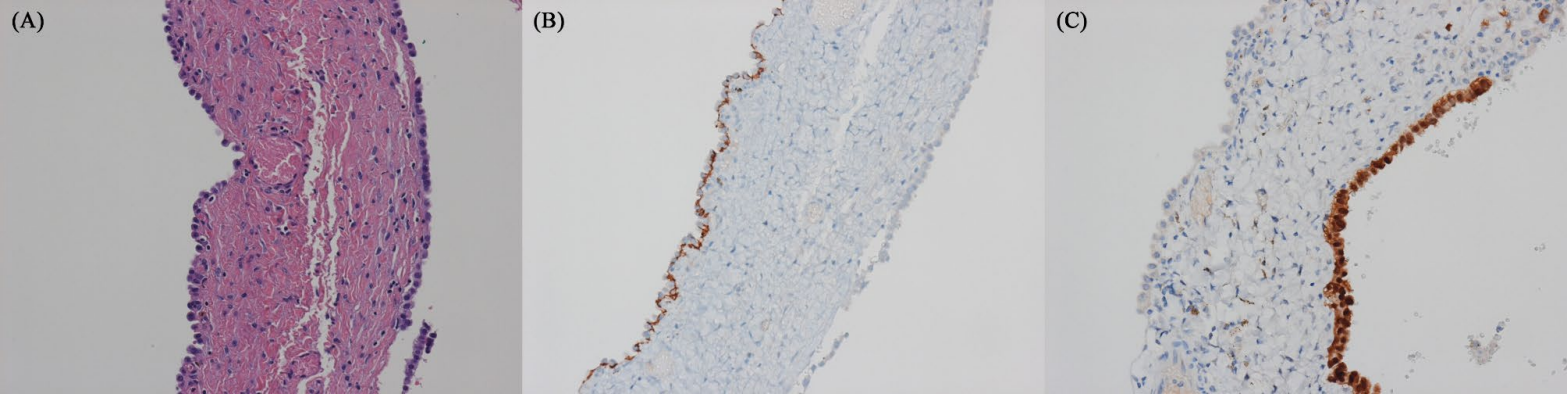
The left part displays atypical cells with occasionally prominent nucleoli and hobnail morphology (MOC-31(+), Calretinin(-)). The right part exhibited flat cuboidal cells, suggesting reactive mesothelial cells (MOC-31(-), Calretinin(+)). **(A)** H&E staining (x200), **(B)** MOC-31 staining (x200), and **(C)** Calretinin staining (x200). H&E, hematoxylin and eosin; MOC-31, anti-epithelial related antigen

To preparing this case report, we carefully reviewed all the slides and found a case of overdiagnosis. The pathologist initially observed atypical cells with occasional prominent nucleoli and hobnail morphology (MOC-31(+), Calretinin(-)). Their growth pattern along the cyst wall and morphology raised concerns about metastatic adenocarcinoma. However, upon reviewing the entire slides, it was more reasonable to consider the cells as reactive alveolar cells because of their uniformly enlarged nonoverlapping vesicular nuclei with regular and smooth nuclear membranes and a single prominent nucleolus. These findings were consistent with subpleural bullous emphysema (Fig. 3).

## Discussion and conclusions

The formation of giant emphysematous bullae stems from the inflammatory destruction and loss of elasticity of small alveolar walls, leading to the coalescence of large air-filled bullae [7]. Radiographically, giant bullous emphysema is characterized by bullae occupying at least one-third of the hemithorax, present in one or both upper lobes [8]. This condition develops due to chronic inflammation of distal airspaces, resulting in emphysematous destruction of the lung parenchyma, subsequent breakdown of the alveolar wall, and permanent enlargement of airspaces [8]. Patients with this condition have a long history of cigarette smoking, marijuana use, and COPD [2, 3]. More recently, HIV infection, Williams-Beuren syndrome, Ehlers-Danlos syndrome, and sarcoidosis have also been identified as additional risk factors [3, 5, 9].

There are reports of an association between giant bullous emphysema and lung cancer. Emphysema is an independent risk factor for lung cancer, with multiple potential pathways implicated in this association, all involving chronic inflammation, anomalous cellular repair, and genetic polymorphisms triggering carcinogenesis [4]. Although the mechanism of carcinogenesis in patients with pulmonary bullous disease remains uncertain, various theories have been proposed. Scar cancer, acquired from repeated inflammatory processes causing the formation of fibrous scar tissue around bullae, as well as impaired ventilation facilitating carcinogen deposition, could lead to metaplastic transformation of epithelial cells within bullae [10]. Additionally, carcinogens may inhibit anti-elastase enzymes, leading to the destruction of the interalveolar septa and subsequent bulla formation [11].

Alveoli are usually lined by type I pneumocytes, but they can also be populated by hyperplastic type II pneumocytes under certain conditions such as diffuse alveolar damage, radiation or drug-related toxicity, infection and various inflammatory or fibrosing conditions. The prominence of hyperplastic type II pneumocytes, as opposed to the typically inconspicuous type I pneumocytes, may raise concerns about adenocarcinoma with a lepidic pattern. The atypical appearance of these cells, especially in organizing diffuse alveolar damage, can be quite pronounced. This atypical appearance, referred to as “too atypical to be malignant”, contrasts with the relatively bland presentation of many adenocarcinoma with a lepidic pattern. Findings related to the underlying condition, such as acute lung injury, fibrosis, or inflammation, usually not only coexist with but also often overshadow reactive pneumocyte hyperplasia [6].

In this case, we initially diagnosed giant bullae as resulting from metastatic adenocarcinoma from lung cancer in the RML, invading the visceral pleura. However, upon reviewing the slides, we found that there was an overdiagnosis of reactive pneumocytes as metastatic adenocarcinoma. This type of diagnostic error can occur because of the similarities in the cellular and morphological features of reactive pneumocytes and malignant cells. The patient displayed multiple tiny nodules and giant bullous emphysema in the contralateral lung, making confirming their association with metastatic adenocarcinoma difficult. It was deemed necessary to monitor the changes in the tiny nodules and remaining giant.

Overdiagnosing reactive pneumocytes as metastatic adenocarcinoma can result in unnecessary treatments and complications. Accurate differentiation between reactive changes and malignancy is crucial to avoid overtreatment. This case highlights the importance of thorough histopathological evaluation and cautious interpretation of atypical cellular morphology.

## Data Availability

No datasets were generated or analysed during the current study.

## Abbreviations

AJCC: American Joint Committee on Cancer
COPD: Chronic obstructive pulmonary disease
CT: Computed tomography
DLCO: Diffusing capacity of the lungs for carbon monoxide
ECOG: Eastern Cooperative Oncology Group
FEV1: Forced expiratory volume in the first second
FVC: Forced vital capacity
HIV: Human immunodeficiency virus
PET-CT: Positron emission tomography-computed tomography
PFT: Pulmonary function test
POD: Postoperative day
PTFE: Polytetrafluoroethylene
RLL: Right lower lobe
RML: Right middle lobe
RUL: Right upper lobe
SUVmax: Maximum standardized uptake value
